# Synergistic Effect of Bypassing Agents and Sequence Identical Analogue of Emicizumab and Fibrin Clot Structure in the In Vitro Model of Hemophilia A

**DOI:** 10.1055/s-0040-1710032

**Published:** 2020-07-21

**Authors:** Yanan Zong, Aleksandra Antovic, Nida Mahmoud Hourani Soutari, Jovan Antovic, Iva Pruner

**Affiliations:** 1 Department of Molecular Medicine and Surgery, Clinical Chemistry and Coagulation, Karolinska Institutet, Stockholm, Sweden; 2 Department of Medicine, Unit of Rheumatology, Karolinska Institutet and Academic Specialist Center, Center for Rheumatology, Stockholm Health Services, Stockholm, Sweden; 3 Department of Clinical Chemistry, Karolinska University Hospital, Stockholm, Sweden

**Keywords:** emicizumab, bypassing agents, fibrin clot, hemophilia A

## Abstract

Development of inhibitors to factor VIII (FVIII) occurs in approximately 30% of severe hemophilia A (HA) patients. These patients are treated with bypassing agents (activated prothrombin complex concentrate [aPCC] and recombinant activated FVII-rFVIIa). Recently, a bispecific FIX/FIXa- and FX/FXa-directed antibody (emicizumab) has been approved for the treatment of HA patients with inhibitors. However, the data from clinical studies imply that coadministration of emicizumab and bypassing agents, especially aPCC, could have a thrombotic effect.

This study was aimed to address the question of potential hypercoagulability of emicizumab and bypassing agents' coadministration, we have investigated fibrin clot formation and structure in the in vitro model of severe HA after adding sequence-identical analogue (SIA) of emicizumab and bypassing agents.

Combined overall hemostasis potential (OHP) and fibrin clot turbidity assay was performed in FVIII-deficient plasma after addition of different concentrations of SIA, rFVIIa, and aPCC. Pooled normal plasma was used as control. The fibrin clots were analyzed by scanning electron microscopy (SEM).

OHP and turbidity parameters improved with the addition of aPCC, while therapeutic concentrations of rFVIIa did not show substantial improvement. SIA alone and in combination with rFVIIa or low aPCC concentration improved OHP and turbidity parameters and stabilized fibrin network, while in combination with higher concentrations of aPCC expressed hypercoagulable pattern and generated denser clots.

Our in vitro model suggests that combination of SIA and aPCC could potentially be prothrombotic, due to hypercoagulable changes in fibrin clot turbidity and morphology. Additionally, combination of SIA and rFVIIa leads to the formation of stable clots similar to normal fibrin clots.

## Introduction

Hemophilia A (HA) is a bleeding disorder characterized by a deficiency of clotting factor VIII (FVIII). Around 50% of HA patients are suffering from severe HA, defined as less than 1% residual FVIII activity. 1 2 These patients experience severe bleeding episodes from early childhood, and without appropriate treatment, repeated bleeding episodes lead to irreversible hemoarthropathy. 3

Standard treatment for HA includes regular prophylaxis or on-demand treatment with recombinant or plasma-derived FVIII. 4 The standard prophylactic regimen consists of intravenous administration of FVIII thrice per week or every other day, either by self-infusion or in a specialized institution, but the burden of frequent therapy requirements is one of the reasons for nonadherence to prophylaxis. Additionally, around 30% of severe HA patients develop inhibitors, neutralizing alloantibodies to FVIII that interfere with FVIII replacement therapy, thus leaving the patients vulnerable to bleeding. 5 6 The therapeutic options for inhibitor patients are limited: either to try to eradicate inhibitors by inducing immune tolerance, which was proven effective in 70% of patients, 7 or treatment with bypassing agents. The most commonly used bypassing agents are activated prothrombin complex concentrate (aPCC, FVIII-bypassing agent, FEIBA NF; Shire, MA, United States) and recombinant activated FVII (rFVIIa, NovoSeven; Novo Nordisk, NJ, United States). 8 9 While proven to reduce bleeding when used for primary or secondary prophylaxis, both aPCC and rFVIIa do not eliminate bleeding or successfully control acute bleeds in all patients with inhibitors. 10 11

There is several nonfactor products that are under development, as there is a need for an effective treatment for inhibitor HA patients. 12 Emicizumab (HEMLIBRA, ACE910; Chugai Pharmaceutical Co., Tokyo, Japan) has been licensed by the U.S. Food and Drug Administration and represents an alternative option for patients with inhibitors. 13 Emicizumab is a recombinant humanized monoclonal bispecific antibody that binds to and bridges factor IXa (FIXa) and factor X (FX), acting as FVIII-mimetic agent. 14 In HAVEN 1 study that included 102 patients with HA and FVIII inhibitors, emicizumab prophylaxis led to 87% reduction of annualized bleeding rate compared with no prophylaxis. 15 Unlike FVIII concentrate and bypassing agents, emicizumab lacks the inherent activation and inactivation which raises the question of product safety, especially when breakthrough bleeding episodes during emicizumab treatment occur. 4 In those cases, patients require treatment with bypassing agents, but there could be an additional thrombotic risk associated with combining emicizumab with bypassing agents. Indeed, HAVEN 1 study reported two patients with venous thrombosis (DVT) and three patients with thrombotic microangiopathy (TMA) who received emicizumab and aPCC, and no complications were reported when patients with inhibitors received emicizumab and rFVIIa. 15 A recent in vitro study demonstrated that there is a 17-fold increase in peak thrombin generation for coadministration of aPCC and a 1.8-fold increase for coadministration of rFVIIa over SIA alone. 16

To address the question of potential hypercoagulability of emicizumab and bypassing agents' coadministration, we have investigated fibrin clot formation and structure in the in vitro model of severe HA after adding different concentrations of SIA and bypassing agents. We performed some of the global hemostasis assays to assess the parameters of fibrin clot formation and investigated the quality of the fibrin network.

## Materials and Methods

### Materials

FVIII-deficient plasma derived from congenital factor VIII-deficient donors and pooled normal plasma were purchased from George King Bio-Medical (Overland Park, Kansas, United States). SIA, aPCC, and rFVIIa were obtained from Shire (Vienna, Austria). SIA was synthesized based on the amino acid sequence published for emicizumab, as previously described. 16 For overall hemostasis potential, thrombin was purchased from Sigma-Aldrich (St. Louis, Missouri, United States), t-PA was from Boehringer Ingelheim (Ingelheim am Rhein, Germany) and Phospholipid Reagent-TGT was purchased from Rossix AB (Mölndal, Sweden). Glutaraldehyde for fibrin clot fixation was purchased from SERVA Electrophoresis GmbH (Heidelberg, Germany).

### Methods

### Overall Hemostasis Potential (OHP) Assay and Fibrin Clot Turbidity Assay

Clotting was initiated in 130 µL of FVIII-deficient plasma with the addition of 34 mmol/L of CaCl _2_ , 0.25 mmol/L of Phospholipid Reagent-TGT, and 0.04 U/mL of thrombin in presence/absence of t-PA (300 ng/mL). SIA was added in the volume of 10µL alone (final concentrations 10, 60, 200, and 600 nM, equivalent to 1.5, 9, 30, and 90 μg/mL, respectively) or in combination with aPCC (final concentrations 25, 50, 250, 500, and 1,000 mU/mL) or rFVIIa (final concentrations 0.88, 1.75, and 5.25 µg/mL, respectively). The concentration ranges of products are chosen to correspond to the therapeutic concentrations, as well as sub- and supratherapeutic concentrations to additionally explore their individual and combined effect. Pooled normal plasma was used as a control. All reactions were performed in triplicate. Clot formation and lysis were monitored by measuring the absorbance at 405 nm for 1 hour in every 12 seconds. The area under the curve (AUC) was calculated by summation of the Abs values (Abs-sum). 17 The overall hemostasis potential (OHP) was calculated as the summation of the Abs values under the fibrin aggregation curve when t-PA was added in the reaction mix, while overall coagulation potential (OCP) was calculated as the summation of Abs values under the fibrin aggregation curve without t-PA. Overall fibrinolysis potential (OFP) was calculated using the equation: OFP = ([OCP − OHP)/OCP] × 100%.

Fibrin clot formation parameters were calculated from OCP curve, as described by Gidley et al, lag time-time point at which exponential growth of the absorbance occurs, the maximum Abs-median value of three consecutive points where the curve reached plateau less the lag turbidity, clotting rate slope of the line fitted from point of the start of the curve exponential growth to point of the plateau, time to plateau time at which maximum Abs is reached, and slope-time duration of the curve exponential growth. 18

### Scanning Electron Microscopy of Fibrin Clots

After the OHP assay was finished, 96 well plates with fibrin clots in the wells without added t-PA were kept in a humid chamber for 1 hour to stabilize the fibrin structure. Fibrin clots were gently removed from plate wells to microtubes with an inoculating loop. The clots were washed with PBS (3 × 5 minute), fixed in 2.5% glutaraldehyde and stored at 4°C until analysis. The fixed clots were washed in MilliQ water twice for 5 minutes, dehydrated serially in 70, 95, and 100% ethanol for 10 minutes, and in acetone twice for 10 minutes. The dehydrated clots were critical point dried using carbon dioxide (Leica EM CPD 030), and mounted on aluminum stubs using silver paint and finally sputter-coated with platinum (Quorum Q150T ES, Quorum, East Sussex, England). Scanning electron microscopy (SEM) images were acquired using an Ultra 55 field emission scanning electron microscope (Zeiss, Germany) at 3 kV and an SE2 detector. Both low- and high-magnification images were acquired at five different locations of each fibrin clot sample. For each sample, 50 individual fibers were randomly selected for thickness measurement using SIS iTEM software (FEI Company, The Netherlands).

### Statistical Analysis

Descriptive statistics were used to analyze the results of this study. All values are presented as mean ± standard deviation (SD).

## Results

### Overall Hemostasis Potential Assay

**Fig. 1:**
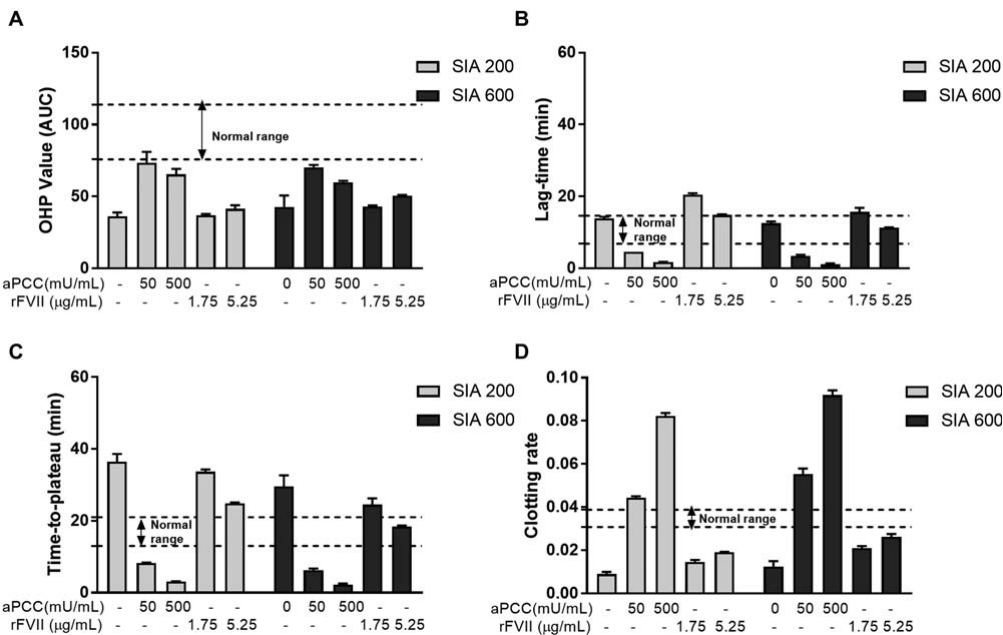
Chosen parameters of OHP ( **A** ) and fibrin clot turbidity assay ( **B–D** ). OHP: overall hemostasis assay presented as the area under the curve (AUC). Normal range: reference range presented as a minimum and maximum value measured in pooled normal plasma (PNP). SIA: sequence identical analogue of emicizumab (200 and 600 nM). aPCC, activated prothrombin complex concentrate (50 and 500 mU/mL); OHP, overall hemostasis potential; rFVIIa, recombinant activated factor VII (1.75 and 5.25 μg/mL).

Fibrin aggregation was monitored after the addition of SIA, aPCC, and rFVIIa as a single agent and in the combination of various concentrations of SIA and aPCC/rFVIIa to FVIII-deficient plasma ( Table 1 ). The normal ranges were established from repeated testing of pooled normal plasma (PNP) and defined as maximum and minimum values of OHP and fibrin clot turbidity assay parameters ( Fig. 1 ).

**Table 1:** OHP and turbidity assay parameters

Condition	OCP (AUC)	OHP (AUC)	lag time (min)	Time to peak (min)	Slope (clotting rate)
Pooled normal plasma	239 ± 17	92.2 ± 13.2	10 ± 3	16 ± 2	0.03 ± 0.003
FVIII-deficient plasma	ND	ND	ND	ND	ND
SIA (600 nM)	189 ± 17	42.8 ± 7.8	13 ± 0.5	30 ± 3.1	0.012 ± 0.0025
SIA (200 nM)	165 ± 15	36.3 ± 2.6	14 ± 0.5	36 ± 2.1	0.009 ± 0.001
SIA (60 nM)	125 ± 1	29.6 ± 2.7	16 ± 0.5	48 ± 2.9	0.006 ± 0.0001
SIA (10 nM)	ND	ND	ND	ND	ND
aPCC (1,000 mU/mL)	258 ± 6	74.5 ± 5.1	5 ± 0.7	12 ± 0.5	0.03 ± 0.0019
aPCC (500 mU/mL)	254 ± 3	73.0 ± 2.1	8 ± 0.2	17 ± 0.1	0.02 ± 0.0002
aPCC (250 mU/mL)	210 ± 14	58.9 ± 2.5	11 ± 1.2	26 ± 2.1	0.01 ± 0.0009
aPCC (50 mU/mL)	ND	ND	ND	ND	ND
aPCC (25 mU/mL)	ND	ND	ND	ND	ND
aPCC (1,000 mU/mL) + SIA (600 nM)	157 ± 5	58.3 ± 1.6	0.7 ± 0.1	1.9 ± 0.1	0.08 ± 0.005
aPCC (500 mU/mL) + SIA (600 nM)	170 ± 8	59.9 ± 1.1	1.1 ± 0.3	2.3 ± 0.3	0.09 ± 0.002
aPCC (250 mU/mL) + SIA (600 nM)	188 ± 3	63 ± 0.4	1.8 ± 0.2	3.3 ± 0.2	0.08 ± 0.0021
aPCC (50 mU/mL) + SIA (600 nM)	224 ± 5	70.2 ± 1.7	3.5 ± 0.3	6.2 ± 0.5	0.05 ± 0.002
aPCC (25 mU/mL) + SIA (600 nM)	239 ± 5	74.1 ± 4.5	4.8 ± 0.2	8.7 ± 0.3	0.04 ± 0.002
aPCC (1,000 mU/mL) + SIA (200 nM)	166 ± 10	66.2 ± 6.4	0.9 ± 0.3	2.1 ± 0.3	0.09 ± 0.002
aPCC (500 mU/mL) + SIA (200 nM)	188 ± 8	65.5 ± 3.7	1.7 ± 0.1	3.1 ± 0.1	0.08 ± 0.001
aPCC (250 mU/mL) + SIA (200 nM)	206 ± 10	73.9 ± 14.5	2.3 ± 0.4	4.0 ± 0.4	0.07 ± 0.002
aPCC (50 mU/mL) + SIA (200 nM)	240 ± 7	73.6 ± 7.5	4.0 ± 0	8.3 ± 0.1	0.04 ± 0.001
aPCC (25 mU/mL) + SIA (200 nM)	253 ± 7	74.4 ± 4.9	5.9 ± 0.6	10.8 ± 0.6	0.04 ± 0.003
rFVIIa (5.25 µg/mL)	37 ± 2	1.3 ± 0.5	13 ± 0.1	49 ± 0.8	0.01 ± 0.0004
rFVIIa (1.75µg/mL)	ND	ND	ND	ND	ND
rFVIIa (0.88 µg/mL)	ND	ND	ND	ND	ND
rFVIIa (5.25 µg/mL) + SIA (600 nM)	226 ± 7	50.4 ± 0.6	11 ± 0.1	18 ± 0.3	0.03 ± 0.001
rFVIIa (1.75µg/mL) + SIA (600 nM)	191 ± 8	43 ± 0.8	16 ± 1.1	24 ± 1.8	0.02 ± 0.003
rFVIIa (0.88 µg/mL) + SIA (600 nM)	172 ± 8	40.3 ± 1.8	17 ± 2	27 ± 2.8	0.02 ± 0.002
rFVIIa (5.25 µg/mL) + SIA (200 nM)	200 ± 4	41.4 ± 2.5	15 ± 0.2	25 ± 0.35	0.02 ± 0.0002
rFVIIa (1.75µg/mL) + SIA (200 nM)	164 ± 3	36.9 ± 0.9	20 ± 0.4	34 ± 0.64	0.02 ± 0.001
rFVIIa (0.88 µg/mL) + SIA (200 nM)	136 ± 14	34.3 ± 4.8	24 ± 1.9	37 ± 1.13	0.01 ± 0.0004

Abbreviations: aPCC, activated prothrombin complex concentrate; ND, nondetectable; OCP, area under the curve (AUC) derived from the overall coagulation potential curve; OHP: AUC derived from overall hemostasis potential curve; rFVIIa, recombinant activated factor VII; SIA, sequence identical analogue of emicizumab.

Note: All values are presented as mean ± standard deviation.

We observed no fibrin aggregation in FVIII-deficient plasma when only a low amount of thrombin and calcium were added. There was a dose-dependent increase of OCP and OHP parameters after addition of SIA ( Table 1 ). However, significant fibrin aggregation was not observed after addition of 10 nM of SIA, due to the delayed onset of aggregation reaction. None of the concentrations of SIA restored the values of OCP and OHP parameters to the values measured for PNP.

Addition of higher doses of aPCC (250, 500, and 1,000 mU/mL) resulted in fibrin aggregation levels similar or higher than that in PNP ( Table 1 , OCP parameter), while the fibrin aggregation onset was delayed significantly when lower doses (25 and 50 mU/mL) were used, thus making the calculation of all OHP assay parameters impossible. Addition of rFVIIa to FVIII-deficient plasma produced low levels of fibrin aggregation with the highest concentration used, while no measurable fibrin aggregation was detected when clinically relevant (1.75 µg/mL corresponding to 120 µg/kg) or lower dose of rFVIIa was used.

Combination of SIA and aPCC did improve OHP assay parameters, but we did not detect fibrin aggregation levels higher than a normal range that would reflect hypercoagulable state ( Table 1 ; Fig. 1A ). Combination of SIA and rFVIIa did lead to a slight improvement of OHP assay parameters ( Table 1 ; Fig. 1A ).

We were not able to measure OFP parameter in FVIII-deficient plasma, due to abnormal fibrin aggregation and clot lysis. Addition of aPCC, SIA and a higher concentration of rFVIIa leads to improved fibrin aggregation, making the measurement of clot lysis possible. None of the single agents used restores the values of OFP parameter to the value measured for PNP. The combination of aPCC and SIA leads to decrease of OFP values to the values comparable to PNP. The combination of rFVIIa and SIA does not lead to a further decrease in OFP values ( Supplementary Table S1 ).

### Fibrin Clot Turbidity Parameters

To further evaluate whether SIA in combination with bypassing agents could exert hypercoagulable state through the formation of dense fibrin clots, we calculated parameters of clot turbidity assay. Similar to the results of the OHP assay, there was a dose-dependent change of all measured parameters ( Table 1 ; Supplementary Table S1 ). Addition of 200 and 600 nM of SIA leads to lag times that were within the defined normal range, time to plateau (TTP) was prolonged and the clotting rate was slower compared with PNP values ( Fig. 1B–D ). Slope time was significantly prolonged, while we detected no significant changes in maximum Abs ( Supplementary Table S1 ). However, the addition of 50 mU/mL of aPCC together with both concentrations of SIA leads to a drastic change of fibrin clot turbidity parameters. Lag time was 2.5-fold shortened when compared with normal range, TTP was two-fold shorter, while the somewhat faster clotting rate was observed. This effect was even more prominent when a higher concentration of aPCC (500 mU/mL) was used ( Fig. 1B–D ).

On the other hand, similarly to the results of the OHP assay, combination of SIA and rFVIIa did improve turbidimetric parameters, but not to the extent comparable to values measured for pooled normal plasma.

### Scanning Electron Microscopy

The representative fibrin clots formed with the following concentrations of SIA and bypassing agents during the OHP assay were chosen for further SEM analysis; PNP, FVIII-deficient plasma, SIA (200 and 600nM), aPCC (50 and 500 mU/mL), rFVIIa (1.75 and 5.25µg/mL), and the corresponding combinations of SIA + aPCC and SIA + rFVIIa. The measured mean fiber thickness is given in Table 2 .

**Table 2:** Fiber thickness of the representative fiber clots

Condition	Mean fiber thickness ± SD
Pooled normal plasma	160 ± 63
FVIII-deficient plasma	ND
SIA (600 nM)	155 ± 78
SIA (200 nM)	171 ± 98
aPCC (500 mU/mL)	150 ± 41
aPCC (50 mU/mL)	220 ± 123
aPCC (500 mU/mL) + SIA (600 nM)	81 ± 22
aPCC (500 mU/mL) + SIA (200 nM)	92 ± 24
aPCC (50 mU/mL) + SIA (600 nM)	96 ± 26
aPCC (50 mU/mL) + SIA (200 nM)	111 ± 28
rFVIIa (5.25 µg/mL)	ND
rFVIIa (1.75µg/mL)	ND
rFVIIa (5.25 µg/mL) + SIA (600 nM)	135 ± 68
rFVIIa (5.25 µg/mL) + SIA (200 nM)	129 ± 55
rFVIIa (1.75µg/mL) + SIA (600 nM)	182 ± 77
rFVIIa (1.75µg/mL) + SIA (200 nM)	132 ± 100

Abbreviations: aPCC, activated prothrombin complex concentrate; ND, nondetectable; rFVIIa, recombinant activated factor VII; SD, standard deviation; SIA, sequence identical analogue of emicizumab.

Note: Data are presented as mean ± SD.

**Fig. 2:**
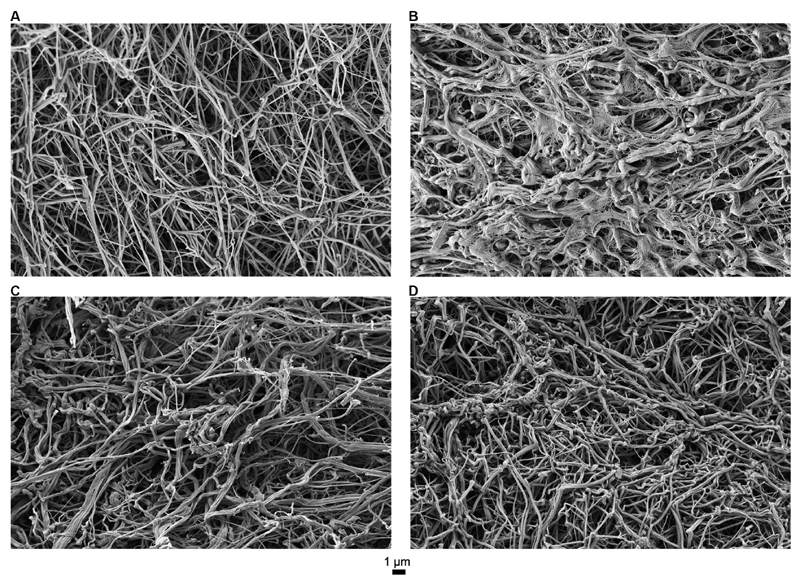
Representative scanning electron microscopy images of fibrin clots. ( **A** ) Pooled normal plasma; ( **B** ) FVIII-deficient plasma; ( **C** ) SIA 200 nM; ( **D** ) SIA 600 nM. F, factor; SIA, sequence-identical analogue.

Fibrin clot formed from the PNP sample had thin and smooth fibers with characteristic normal branching and small pores ( Fig. 2A ), while fibrin clot formed from FVIII-deficient plasma was composed of relatively thick and short fibers with no structured network. The network density was highly heterogeneous with large pores and occasional structures reminiscent of spider webs, presumably forming due to hindered cross-linking of fiber polymers ( Fig. 2B ). After addition of SIA, there was a significant improvement of fibrin structure. Addition of 200 nM SIA leads to the formation of slightly thicker fibers with larger pores in a not entirely structured fibrin network ( Fig. 2C ), while the addition of 600 nM SIA lead to the formation of fibrin fibers with similar thickness as those in the PNP sample ( Fig. 2D ).

**Fig. 3:**
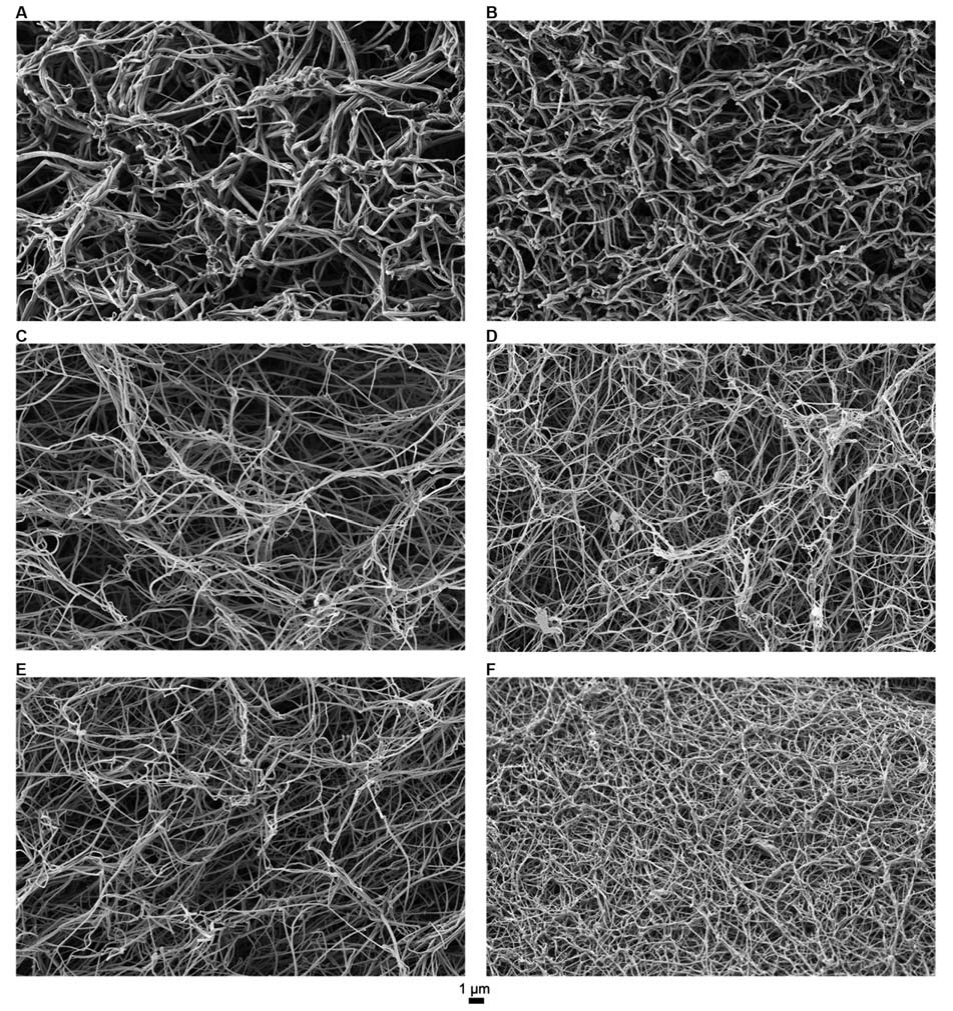
Representative scanning electron microscopy images of fibrin clots. ( **A** ) aPCC (50 mU/mL); ( **B** ) aPCC (500 mU/mL); ( **C** ) aPCC (50 mU/mL) + SIA (200 nM); ( **D** ) aPCC (500 mU/mL) + SIA (200 nM); ( **E** ) aPCC (50 mU/mL) + SIA (600 nM); ( **F** ) aPCC (500 mU/mL) + SIA (600 nM). aPCC, activated prothrombin complex concentrate; SIA, sequence-identical analogue.

Addition of 50 mU/mL of aPCC to FVIII-deficient plasma leads to the formation of fibrin clots with smooth and increasingly thicker fibers, although the fibrin network is not completely structured and large pores were observed ( Fig. 3A ). On the other hand, the addition of a higher concentration of aPCC (500 mU/mL) leads to the formation of fibrin clot with the structure similar to the PNP clots ( Fig. 3B ). Addition of 200 and 600 nM of SIA together with 50 mU/mL of aPCC leads to the formation of clots with prominently thin and smooth fibers with regular branching and increased clot density ( Fig. 3C and E ). This effect is even more noticeable when SIA is combined with 500 mU/mL of aPCC. There is an up to a two-fold decrease in fiber thickness compared with PNP clot with highest concentrations of aPCC and SIA combined ( Table 2 ), with dense and homogenous fibrin ( Fig. 3D and F ).

**Fig. 4:**
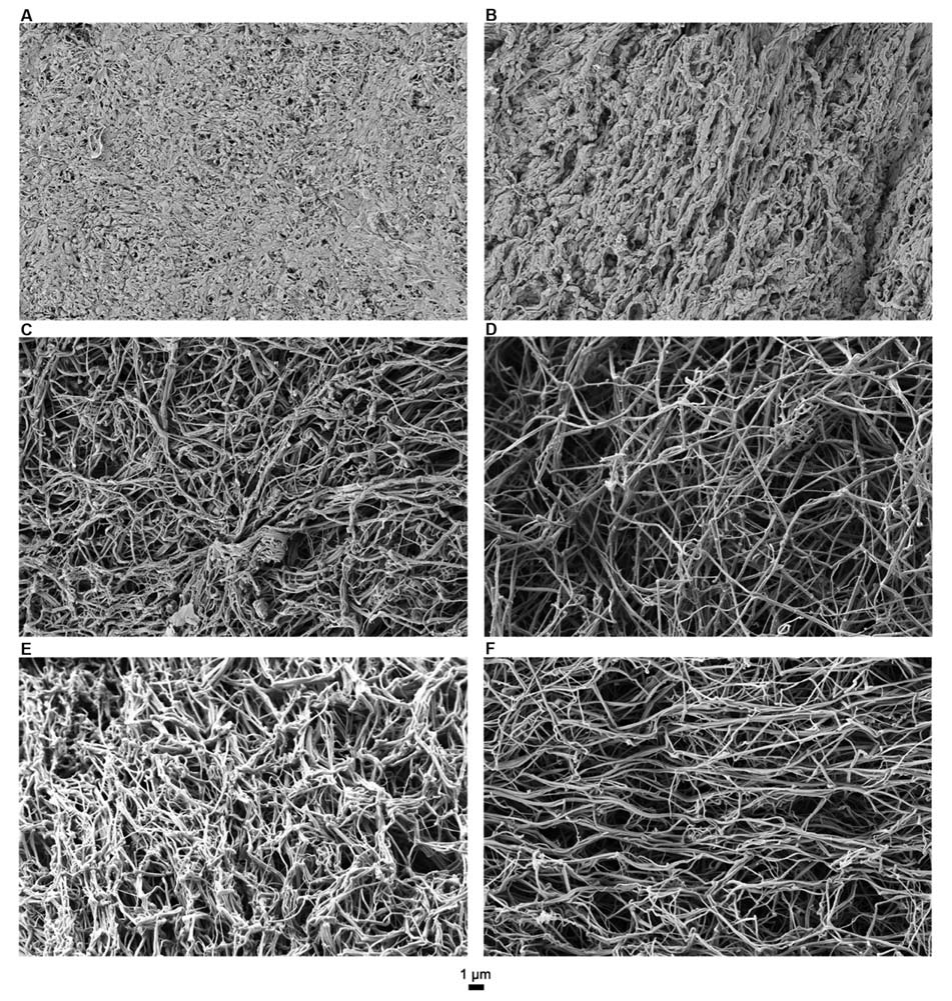
Representative scanning electron microscopy images of fibrin clots. ( **A** ) rFVIIa (1.75µg/mL); ( **B** ) rFVIIa (5.25 µg/mL); ( **C** ): rFVIIa (1.75 µg/mL) + SIA (200 nM); ( **D** ) rFVIIa (5.25 µg/mL) + SIA (200 nM); ( **E** ) rFVIIa (1.75 µg/mL) + SIA (600 nM); ( **F** ) rFVIIa (5.25 µg/mL) + SIA (600 nM). rFVIIa: recombinant activated factor VII; SIA, sequence-identical analogue.

Addition of rFVIIa leads to the formation of completely unstructured clots. Individual fibers could not be discerned and measured ( Fig. 4A and B ). Addition of 200 and 600 nM of SIA in combination with 1.75 µg/mL of rFVIIa leads to the formation of clots with smooth and thick fibers, randomly interconnected to bundles with sporadic spider web structures observed also in FVIII-deficient clots. The pores are large and the branching is irregular, indicating loose structures ( Fig. 4C and E ). On the other hand, SIA in combination with 5.25 µg/mL of rFVIIa improves the quality of fibrin clot significantly. Fibers are thin and smooth with branching similar to the one observed in PNP clots ( Fig. 4D and F ).

## Discussion

This is to our knowledge, the first study investigating the effect of different combinations of SIA and bypassing agents on the fibrin formation and fibrin morphology. To explore the potential hypercoagulable effect of emicizumab and bypassing agents' combination, we performed OHP and fibrin clot turbidity assay and analyzed the fibrin network quality of chosen SIA-bypassing agents' combinations by SEM. Our results have shown that there is a potential combined effect of SIA and aPCC, reflected through the significantly shortened onset of fibrin aggregation (lag time) and time to peak, as well as increased clotting rate, that could lead to the development of hypercoagulable states.

There are several advantages that are provided by the use of emicizumab. First, emicizumab prophylaxis significantly reduced the annual bleeding rate of patients with HA with or without inhibitors, and second, the mode of administration and frequency (s.c every 1 or 2 weeks) makes the use of emicizumab less burdensome for patients. 19 However, if breakthrough bleedings occur in some of the patients with inhibitors undergoing emicizumab therapy, bypassing agents must be administered to control the bleeding episodes. The long half-life of emicizumab means that these patients may not be able to wait for emicizumab clearance before administrating aPCC or rFVIIa.

The HAVEN 1 trial reported patients developing thrombotic events (DVT and TMA) when aPCC was administered for control of breakthrough bleeds at doses averaging more than 100 U/kg/day for more than 1 day, while no thrombotic events occurred when rFVIIa was administered. 15 Previous in vitro study which measured thrombin generation at clinically relevant doses of sequence identical analogue of emicizumab in combination with aPCC and rFVIIa revealed significantly increased thrombin levels when SIA is combined with aPCC, suggesting that care should be taken when patients receiving emicizumab are treated with aPCC to control breakthrough bleedings. Additional clot formation analysis employing total thrombus formation analysis system (T-TAS) and rotational thromboelastometry (ROTEM) showed that clot formation time (CFT) of SIA–aPCC is two- to eightfold lower compared with healthy controls. 16

We did not observe changes in OCP, OHP, and OFP values that could point to the potential hypercoagulable effect of SIA and bypassing agents. These values did not reach the normal range of pooled normal plasma, even though we did observe a dose-dependent change for all used combinations of agents. While previous studies did demonstrate the sensitivity of OHP assay for both hypercoagulable and hypocoagulable states, 20 we were not able to clearly demonstrate the change from hypocoagulable to hypercoagulable phenotype for SIA-bypassing agents' combination.

On the other hand, calculation of fibrin clot turbidity parameters revealed drastic changes that could drive hypercoagulation of SIA–aPCC combination but not SIA–rFVIIa combination. Even at low concentration, addition of aPCC to 200 and 600 nM of SIA lead to decrease of lag time and TTP, while the clotting rate was faster, with this effect being even more clear when the higher concentration was used, suggesting that aPCC could induce hypercoagulable state when added together with SIA, through the velocity of fibrin clot formation. Further, we have also shown there is a change of fibrin clot phenotype when SIA–aPCC combination is used. Fibrin clot obtained from FVIII-deficient plasma had abnormally thick fibers with increased porosity, 21 22 a phenotype that is consistent with low thrombin and fibrin generation. 23 24 SIA and aPCC alone lead to the formation of fibrin clots with the structure and fiber thickness similar to the one observed in normal clots. On the other hand, SIA–aPCC combination leads to the formation of dense clots with thin fibers, a fibrin clot phenotype that is associated with high thrombin levels as a mechanism for a potential increased thrombotic risk. 25 These findings are consistent with a study that showed an increase of thrombin generation in the case of SIA–aPCC combination, through the possibility of SIA to more easily form FX activation complex in the presence of aPCC, as hypothesized by authors. 16 It was also shown that aPCC did not have any effect on clot stability alone, 26 contradictory to our study where aPCC alone did stabilize fibrin clot structure to the extent comparable to normal fibrin clots. Interestingly, the prothrombotic phenotype of the structure of the fibrin clots generated in SIA–aPCC treated FVIII-deficient plasma was not reflected by the decrease of overall fibrinolysis potential, as OFP values were not significantly decreased when compared with pooled normal plasma. It was previously shown that there is a defective down-regulation of the fibrinolytic system by the intrinsic pathway, 27 as low thrombin generation limits activation of tissue factor activated fibrinolysis inhibitor (TAFI). The limitations of our assay do not allow us to explore the specific events of clot lysis, especially TAFI activation, which could explain the discrepancy between fibrin clot structure and OFP value. Additionally, there are no currently available studies that explore fibrinolytic events in patients receiving emicizumab. We speculate that impaired fibrinolysis of FVIII-deficient plasma could be restored to the levels of pooled normal plasma by SIA-aPCC coapplication but not to the levels that could reflect prothrombotic phenotype.

Interestingly, the measurement of fibrin clot turbidity parameters was possible only when the highest doses of rFVIIa were used. Compared with pooled normal plasma, the start of the fibrin aggregation reaction was slightly delayed in rFVIIa treated FVIII-deficient plasma, while TTP was very prolonged with slow clotting rate. The analysis of the fibrin clot structure showed the absence of pores in the clots, while no distinguishable individual fibrin fibers were detected. Wolberg et al 28 showed in the in vitro model of HB that hemophilic clot structure could be partially normalized if clots are formed in the presence of high doses of rFVIIa. However, this result cannot be completely translated to HA. In our previous study, we have shown that clots formed from plasma samples of the patients with HB are less permeable than the clots formed from plasma samples of patients with HA, 29 making the probable effect of rFVIIa in HA model less prominent. Indeed, it was shown in other conditions that the effect of rFVIIa is more directed toward thrombin generation rather than improvement of fibrin clot structure. Tanaka et al 30 have demonstrated using the thromboelastography in healthy volunteers and patients after cardiopulmonary bypass that rFVIIa itself does shorten clotting time (onset of fibrin aggregation) but has no effect on maximal clot firmness. Similar results were obtained by Lak et al 31 who have demonstrated using ROTEM in patients with Glanzmann's thrombasthenia rFVIIa improves clotting time but does not affect clot formation time or maximum clot firmness. Addition of SIA leads to stabilization of fibrin clot parameters, rendering the clotting reaction possible even at low concentrations of rFVIIa. This improvement was also visible in the fibrin clot structure, as unstructured fibrin clots obtained from rFVIIa treated FVIII-deficient plasma did change to fibrin structures similar to normal clots, an effect mostly prominent with a higher concentration of rFVIIa. Our results are in accordance with HAVEN 1 study that did not report any thrombotic events when emicizumab and rFVIIa were used. 15

## Limitations

The major limitation of our study is in vitro nature of the experiments and use of pooled FVIII-deficient plasma as a model for severe HA. With this approach, we were not able to measure individual factors that contribute to differences in fibrin clot formation that happen in the in vivo setting. Additionally, our results indicate that attention should be directed toward the fibrinolytic events in patients receiving emicizumab who are later treated with aPCC for bleeding episodes. Further study should include patients receiving emicizumab as primary therapy, so the other individual parameters that influence the formation of fibrin clot could be taken into account. 21

## Conclusion

In conclusion, our in vitro study gives additional rationale that additional caution should be taken when patients on emicizumab therapy are treated with aPCC for breakthrough bleeds and dose tailoring of this bypassing agent is certainly to be considered for optimal patient outcome and care. Additionally, our study shows that clot stability and structure could be utilized as determinants of therapy efficiency in HA.
