# Supplementary material for: Synergistic Effect of Bypassing Agents and Sequence Identical Analogue of Emicizumab and Fibrin Clot Structure in the In Vitro Model of Hemophilia A
Source: TH Open. 2020 Jul 21;4(2):e94–e103. doi: 10.1055/s-0040-1710032 (PMC7373667; doi:10.1055/s-0040-1710032)
Supplement: Supplementary file 1 — Supplementary Material [file 10-1055-s-0040-1710032-s190057.pdf]

Supplementary Table S1 OHP and turbidity assay parameters

Condition	OFP(%)	Max Abs	Slope time (min)
Pooled normal plasma	61.35 ± 4.95	0.97 ± 0.03	6 ± 0.3
FVIII-deficient plasma	ND	ND	ND
SIA (600 nM)	77.18 ± 5.15	0.92 ± 0.03	17 ± 2.7
SIA (200 nM)	77.96 ± 0.34	0.92 ± 0.03	23 ± 2.0
SIA (60 nM)	76.28 ± 2.41	0.88 ± 0.04	32 ± 2.4
SIA (10 nM)	ND	ND	ND
aPCC (1,000 mU/mL)	71.16 ± 1.96	0.92 ± 0.03	6 ± 0.03
aPCC (500 mU/mL)	71.32 ± 1.01	0.99 ± 0.02	10 ± 0.03
aPCC (250 mU/mL)	71.88 ± 3.05	0.99 ± 0.03	15 ± 1.0
aPCC (50 mU/mL)	ND	ND	ND
aPCC (25 mU/mL)	ND	ND	ND
aPCC (1,000 mU/mL) + SIA (600 nM)	62.8 ± 1.73	0.50 ± 0.01	1.2 ± 0
aPCC (500 mU/mL) + SIA (600 nM)	64.82 ± 1.09	0.52 ± 0.02	1.2 ± 0
aPCC (250 mU/mL) + SIA (600 nM)	66.52 ± 0.27	0.58 ± 0.009	1.47 ± 0.12
aPCC (50 mU/mL) + SIA (600 nM)	68.66 ± 1.20	0.74 ± 0.007	2.73 ± 0.23
aPCC (25 mU/mL) + SIA (600 nM)	69.02 ± 2.32	0.82 ± 0.01	3.93 ± 0.12
aPCC (1,000 mU/mL) + SIA (200 nM)	59.86 ± 5.92	0.51 ± 0.03	1.2 ± 0
aPCC (500 mU/mL) + SIA (200 nM)	64.98 ± 3.40	0.58 ± 0.02	1.4 ± 0
aPCC (250 mU/mL) + SIA (200 nM)	63.89 ± 8.85	0.64 ± 0.04	1.67 ± 0.11
aPCC (50 mU/mL) + SIA (200 nM)	69.25 ± 3.55	0.82 ± 0.02	3.73 ± 0.012
aPCC (25 mU/mL) + SIA (200 nM)	70.59 ± 1.51	0.88 ± 0.04	4.93 ± 0.23
rVIIa (5.25 µg/mL)	96.44 ± 1.46	0.89 ± 0.03	36 ± 0.7
rVIIa (1.75µg/mL)	ND	ND	ND
rVIIa (0.88 µg/mL)	ND	ND	ND
rVIIa (5.25 µg/mL) + SIA (600 nM)	77.66 ± 0.72	0.91 ± 0.02	7 ± 0.4
rVIIa (1.75µg/mL) + SIA (600 nM)	77.49 ± 0.67	0.87 ± 0.01	9 ± 0.6
rVIIa (0.88 µg/mL) + SIA (600 nM)	76.58 ± 1.40	0.82 ± 0.03	10 ± 1.13
rVIIa (5.25 µg/mL) + SIA (200 nM)	79.27 ± 0.98	0.91 ± 0.01	10 ± 0.4
rVIIa (1.75µg/mL) + SIA (200 nM)	77.47 ± 0.75	0.91 ± 0.01	13 ± 0.5
rVIIa (0.88 µg/mL) + SIA (200 nM)	74.87 ± 0.99	0.84 ± 0.04	13 ± 0.8

Abbreviations: aPCC, activated prothrombin complex concentrate; Max Abs, maximum absorbance; ND, nondetectable; OFP, overall fibrinolysis potential; OHP, overall hemostasis potential; rFVIIa, recombinant activated FVII; SIA, sequence identical analogue of emicizumab.
Note: All values are presented as mean ± standard deviation.
